# Identification and characterization of microRNAs from *Phaeodactylum tricornutum *by high-throughput sequencing and bioinformatics analysis

**DOI:** 10.1186/1471-2164-12-337

**Published:** 2011-06-30

**Authors:** Aiyou Huang, Linwen He, Guangce Wang

**Affiliations:** 1 Key Laboratory of Experimental Marine Biology, Institute of Oceanology, Chinese Academy of Sciences (IOCAS), Nanhai Road 7, Qingdao 266071, China; 2 School of Earth Science, Graduate University of Chinese Academy of Sciences, Yuquan Road 19, Beijing 100049, China

## Abstract

**Background:**

Diatoms, which are important planktons widespread in various aquatic environments, are believed to play a vital role in primary production as well as silica cycling. The genomes of the pennate diatom *Phaeodactylum tricornutum *and the centric diatom *Thalassiosira pseudonana *have been sequenced, revealing some characteristics of the diatoms' mosaic genome as well as some features of their fatty acid metabolism and urea cycle, and indicating their unusual properties. To identify microRNAs (miRNAs) from *P. tricornutum *and to study their probable roles in nitrogen and silicon metabolism, we constructed and sequenced small RNA (sRNA) libraries from *P. tricornutum *under normal (PT1), nitrogen-limited (PT2) and silicon-limited (PT3) conditions.

**Results:**

A total of 13 miRNAs were identified. They were probable *P. tricornutum*-specific novel miRNAs. These miRNAs were sequenced from *P. tricornutum *under normal, nitrogen-limited and/or silicon-limited conditions, and their potential targets were involved in various processes, such as signal transduction, protein amino acid phosphorylation, fatty acid biosynthetic process, regulation of transcription and so on.

**Conclusions:**

Our results indicated that *P. tricornutum *contained novel miRNAs that have no identifiable homologs in other organisms and that they might play important regulator roles in *P. tricornutum *metabolism.

## Background

Diatoms are important planktons that are believed to be responsible for one-fifth of the primary productivity on Earth [1,2]. There are two major classes of diatoms, the pennates and the centrics. With their vital role in silica cycling [3,4], the unusual evolutionary position of secondary endosymbiotic origin [5-9], the presence of C_4 _photosynthesis in some species [10], and potential as sources of biodiesel fuel [11], diatoms have attracted increasing attention. As early as 2002, Scala et al. [12] analyzed EST (expression sequence tag) data of the pennate diatom *Phaeodactylum tricornutum *and found that some of its genes were more similar to those of animals than of photosynthetic counterparts, implying an unusual evolutionary history. The genome of *P. tricornutum *and the centric diatom *Thalassiosira pseudonana *have been sequenced, shedding light on significant features of diatom genomes, including the mosaic genome that contains 'animal-like', 'plant-like' and 'bacteria-like' genes, performing fatty acid metabolism in both peroxisomes and mitochondria, and the presence of enzymes necessary for a complete urea cycle [7,13,14]. These characteristics prompted us to hypothesize that the gene expression regulators (e.g. miRNAs) of diatoms may show some different specificity to other photosynthetic organisms.

miRNAs are important post-transcriptional regulators. They regulate gene expression in eukaryotes by targeting mRNAs for translational repression or cleavage [15-17]. It is believed that miRNAs exist extensively in eukaryotes such as animals and plants with high conservation in each kingdom [18,19]. The expression of miRNAs has a spatio-temporal pattern [15,17,20-22] and they influence the transcription and translation of many genes [18]. Generally, their functions involve various processes, including developmental patterning, organ separation, cell differentiation and proliferation, tumor generation, cell death and cell apoptosis, stress resistance, auxin response, fat metabolism and miRNA biogenesis [18]. In higher plants and animals, miRNAs have been extensively studied but rarely so in algae.

*P. tricornutum *is an atypical diatom with a weakly silicified outer shell, and the unusual property of being pleiomorphic with three convertible morphotypes [23] (i.e. oval, fusiform and triradiate), and silicification essentially restricted to one valve of the oval cells [24-28]. With its characteristics of short life-cycle, small genome size and ease of transformation, *P. tricornutum *has become an attractive photosynthetic model [12,14,29,30]. Additionally, being rich in polyunsaturated fatty acid (PUFA), especially in eicosapentaenoic acid (EPA), *P. tricornutum *has been used as a food organism and is considered a potential source of EPA. There have been many studies investigating the factors affecting its cell composition [31-34]. There were reports that microalgae accumulated lipids under nitrogen-limited as well as silicon-limited conditions [35,36], with similar studies conducted on *P. tricornutum *[33,34]. Accumulation of lipids in cells and a significant change in fatty acid composition were observed in *P. tricornutum *under low nitrogen conditions. Using suppression subtractive hybridization technology, Tang et al. separated a number of upregulated genes from *P. tricornutum *under nitrogen starvation, seven of which had high similarity with functional genes related to nitrogen utilization [37]. Studies of lipid metabolism of *P. tricornutum *under silicon-limited conditions are scarce. Notwithstanding, Sapriel et al. identified 223 genes regulated by silicic acid availability, including 13 upregulated and 210 downregulated genes, from *P. tricornutum *under silicon-limited conditions [38]. Interestingly, they also observed some upregulated genes coding for transporters of metabolites related to nitrogen assimilation and transfer from *P. tricornutum *in the complete medium compared to silicon-limited conditions. A previous study on *T. pseudonana *showed that a glutamate acetyltransferase was involved in silicon metabolism [39]. How are these genes regulated? Do miRNAs play a role in *P. tricornutum *nitrogen and silicon metabolism? There have been few studies that address these questions.

In the present study, we constructed small RNA (sRNA) libraries from *P. tricornutum *under normal, nitrogen-limited and silicon-limited conditions and then used high-throughput Solexa technology to deeply sequence the sRNAs. The sequencing data were analyzed and miRNAs were identified from all samples studied.

## Results

### A diverse set of endogenous small RNAs

**Figure 1:**
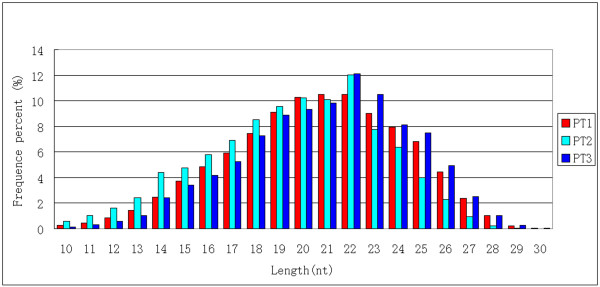
**Length distributions of unique small RNA sequences in *P. tricornutum***. The length occurrence of each unique sequence reads was counted to reflects relative expression level. Only small RNA sequences with length ranged from 10 to 30 nt were considered. Data for different samples were indicated.

**Figure 2:**
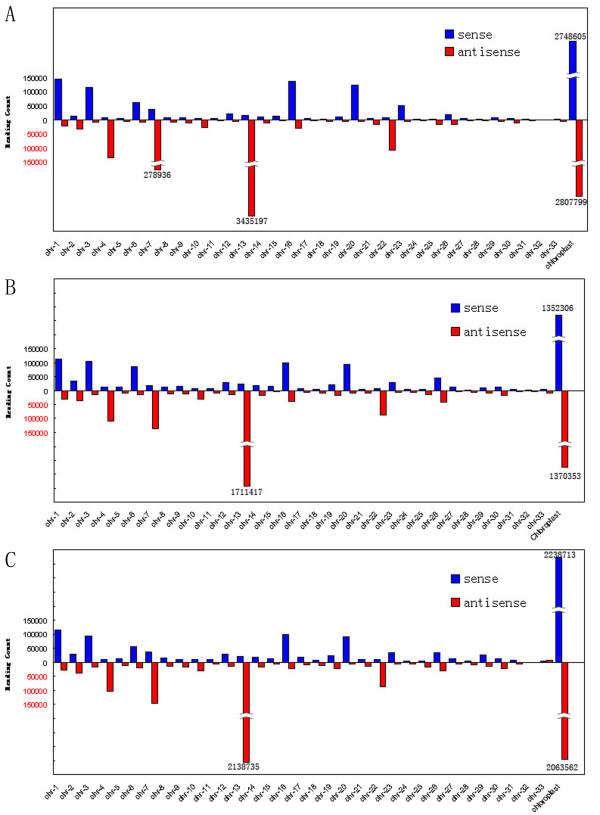
**Small RNA (redundant sequences) distribution across different chromosomes**. Y axis, number of small RNA tags that located on each chromosomes. X axis, chromosomes. Bars above the axis represent matches to the plus strand; bars below the axis represent those to the minus strand. (A) PT1. (B) PT2. (C) PT3.

To determine the likely roles of miRNAs in nitrogen and silicon metabolism in *P. tricornutum*, we constructed and sequenced small RNA libraries from *P. tricornutum *grown in normal (PT1), nitrogen-free (PT2) and silicon-free (PT3) media, respectively. After removing adaptor sequences and filtering out low quality data (see Additional file 1 for flow chart of the procedure for processing of reads), we obtained small RNAs with size range of 10-30 nt, with an enrichment in 20-22 nt (Figure 1). After removing sequences shorter than 18 nt, we obtained 8 924 476, 5 609 466 and 6 982 282 total sequences, representing 718 770, 596 498 and 672 323 unique, although sometimes partially overlapping, clean reads from PT1, PT2 and PT3, respectively (Table 1). Of these unique sequences, about 73% (521 761), 74% (441 959) and 73% (491 748) were only sequenced once. There were 4 105 629, 2 492 000 and 2 908 127 total; and 221 523, 262 038 and 250 371 unique sequences with at least one perfect match in the *P. tricornutum *nuclear genome - whereas 3 076 974, 1 503 395 and 2 410 100 total; and 68 048, 43 151 and 55 321 unique sequences matched the chloroplast genome, in PT1, PT2 and PT3, respectively (Table 1). It was quite unexpected that a majority of sRNAs were located in the minus strand of chromosome 13 and both strands of the chloroplast genome (Figure 2). The usual preference for a U at the 5' - end of plant small RNA sequences [40] was not observed (see Additional file 2 for redundant small RNA nucleotide bias at each position). The four types of bases appeared equally in each locus.

**Table 1:** Total and unique sRNAs in *P. tricornutum*.

			**match genome**^**a**^		**match chloroplast**^**b**^		**Appeared once**^**c**^	
**samples**	**Total sRNA**	**Unique sRNA**	**Total sRNA**	**Unique sRNA**	**Total sRNA**	**Unique sRNA**		

PT1	8924476	718770	4105629	221523	3076974	68048	521761	73%

PT2	5609466	596498	2492000	262038	1503395	43151	441959	74%

PT3	6982282	672323	2908127	250371	2410100	55321	491748	73%

^a^sRNAs that matched nuclear genome.

^b^sRNAs that matched chloroplast genome.

^c^unique sRNAs that appeared once.

All clean reads were annotated according to their identities with non-coding RNAs (Rfam, GenBank), plant miRNAs (miRBase), exon and intron (*P. tricornutum *genome) and siRNAs (Table 2 and Additional file 3). In the case that some sRNA was mapped to more than one category, the following priority rule was adopted: rRNA etc. (in which GenBank > Rfam) > known miRNA > exon > intron [41]. rRNA degraded fragments were the most abundant sequences retrieved from the *P. tricornutum *total sRNA pools, boasting the highest read frequency of all small RNA classes in all the samples: 62.53, 48.29 and 54.96% for PT1, PT2 and PT3, respectively (Table 2 and Additional file 3). Yet in the unique sRNA pools, non-annotated sRNA represented a significant part, with 50.53, 50.61 and 54.98% in PT1, PT2 and PT3, respectively. Homologs of plant known miRNAs accounted for approximately 0.5% of the unique sequences in all the three samples, whereas in total sequences pools, the numbers were approximately 0.6% in PT2 and PT3 and only 0.4% in PT1. sRNAs mapped to exons and introns in either sense or antisense directions also represented a considerable part. The remaining sRNAs were snRNA, snoRNA and tRNA. Common and specific sequences analysis showed that only approximately 15% of the unique sequences were shared by every two samples (Table 3 and Additional file 4), suggesting a diverse set of endogenous small RNAs in *P. tricornutum*.

**Table 2:** Categorization of *P. tricornutum *small RNAs.

	PT1				PT2				PT3			
**Category**	**Unique sRNA**	**Percent (%)**	**Total sRNA**	**Percent (%)**	**Unique sRNA**	**Percent (%)**	**Total sRNA**	**Percent (%)**	**Unique sRNA**	**Percent (%)**	**Total sRNA**	**Percent (%)**

Total	718770	100%	8924476	100%	596498	100%	5609466	100%	672323	100%	6982282	100%

Exon antisense	6399	0.89%	10386	0.12%	12995	2.18%	21742	0.39%	16265	2.42%	29367	0.42%

Exon sense	132234	18.40%	709873	7.95%	157692	26.44%	491026	8.75%	126953	18.88%	468933	6.72%

Intron antisense	738	0.10%	3668	0.04%	1054	0.18%	6759	0.12%	1331	0.20%	5795	0.08%

Intron sense	3244	0.45%	10084	0.11%	3334	0.56%	14835	0.26%	3773	0.56%	28637	0.41%

miRNA	3660	0.51%	36409	0.41%	2821	0.47%	36511	0.65%	3573	0.53%	44646	0.64%

rRNA	187636	26.11%	5580308	62.53%	101013	16.93%	2708557	48.29%	129970	19.33%	3837474	54.96%

siRNA	499	0.07%	1206	0.01%	2032	0.34%	6135	0.11%	2483	0.37%	7836	0.11%

snRNA	395	0.05%	1769	0.02%	358	0.06%	1843	0.03%	384	0.06%	3034	0.04%

snoRNA	147	0.02%	603	0.01%	130	0.02%	858	0.02%	156	0.02%	1381	0.02%

tRNA	20630	2.87%	798584	8.95%	13200	2.21%	445663	7.94%	17783	2.65%	703652	10.08%

non-annotated^a^	363188	50.53%	1771586	19.85%	301869	50.61%	1875537	33.44%	369652	54.98%	1851527	26.52%

^a^Not annotated sRNAs.

**Table 3:** Common and specific small RNAs between every two samples.

	Class	Unique sRNA	Percent (%)	Total sRNA	Percent (%)
PT1_&_PT2	Total_sRNAs	1141621	100.00%	14533942	100.00%

	PT1_&_PT2	173647	15.21%	13293850	91.47%

	PT1_specific	545123	47.75%	741310	5.10%

	PT2_specific	422851	37.04%	498782	3.43%

PT2_&_PT3	Total_sRNAs	1099233	100.00%	12591748	100.00%

	PT2_&_PT3	169588	15.43%	11460968	91.02%

	PT2_specific	426910	38.84%	497883	3.95%

	PT3_specific	502735	45.74%	632897	5.03%

PT1_&_PT3	Total_sRNAs	1190759	100.00%	15906758	100.00%

	PT1_&_PT3	200334	16.82%	14690632	92.35%

	PT1_specific	518436	43.54%	659616	4.15%

	PT3_specific	471989	39.64%	556510	3.50%

### miRNAs in *P. tricornutum*

The identification of a great quantity of small RNAs in *P. tricornutum *prompted us to examine whether some were miRNAs. First we compared all the non-annotated sRNAs with the sequences of animal miRNAs and virus miRNAs available from miRBase (miRBase Sequence Database version 15) [42] to identify homologs of known miRNAs. Then we used the small RNAs with homology to all known miRNAs (including plant, animal and virus miRNAs) and the remaining non-annotated sRNAs to identify candidate known and novel miRNA families in *P. tricornutum*, respectively (see Additional file 1 for flow chart of the procedure for miRNA identification). First we mapped these small RNAs onto the *P. tricornutum *nuclear genome. Then we extracted 300 nt upstream and 300 nt downstream from those loci and examined whether they could form hairpin secondary structures, a character of known plant and animal pre-miRNAs, using criteria developed previously for plant miRNA prediction [43]. Basically, precursors with free energy ≤ -18 kcal/mol checking by Mfold [44,45], ≥ 16 bp and ≤ 4 bulges or asymmetries between miRNA and miRNA*, with miRNA sequence length between 18-25nt and flank sequence length of 20, were considered as potential *P. tricornutum *pre-miRNAs and selected for further analysis. Secondary structural predictions identified a total of 21 small RNA species that were derived from genomic loci whose surrounding sequences had the probability to form hairpin structures that met the requirements as a miRNA precursor. Then we checked for the structure stabilities of these 21 sequences. Among these, five were found to have a P-value lower than 0.05. They were checked for 5' homogeneity using 0.5 as cut off. For those sequences with a P-value above 0.05, a more stringent 5' homogeneity of 0.75 was used. All together we obtained 14 sequences for manually rechecking according to criteria made previously for miRNA identification [46-48]. Finally we determined 13 sequences to be *P. tricornutum *miRNAs. They were submitted to miRBase and named pti-miR5471-5483. Of these 13 small RNAs, seven of pre-miRNA hairpins were supported by EST data.

Each miRNA had a single precursor. The length of pre-miRNA ranged from 101 to 360 nt, with a mean of 235 nt (Table 4, see Additional file 5 for patterns of reads mapped to the pre-miRNAs and Additional file 6 for figures of stem loops for pre-miRNAs). The MFE range was -105 to -26.1 kcal/mol, with a mean of -67.61 kcal/mol. Most pre-miRNAs were located in intergenic regions and the others were mapped to genes of hypothetical protein, probably being mis-annotated.

**Table 4:** Characteristics of *P. tricornutum *pre-miRNA sequences.

miRNA name	**location**^**a**^	**mfe**^**b**^	**len**^**c**^	**P-value**^**d**^	**total reads and 5' homogeneity**^**e**^			**hairpin characteristics **^**f**^	
					**PT1**	**PT2**	**PT3**	**bulges**	**mismatched**

pti-miR5471	chr_1:1604545:1604822:-	-78.8	278	0.172	0/0	9/9	0/0	0	5

pti-miR5472	chr_10:761305:761625:-	-85.9	321	0.355	64/88	23/30	145/213	0	5

pti-miR5473	chr_11:35406:35506:-	-30.4	101	0.019	6/9	0	9/15	0	4

pti-miR5474	chr_15:663006:663165:+	-42.7	160	0.801	22/23	69/71	20/22	0	6

pti-miR5475	chr_18:415320:415541:-	-62	222	0.44	0/0	226/230	383/385	0	4

pti-miR5476	chr_19:299730:299870:-	-52.8	141	0.043	0/0	0/0	9/15	0	3

pti-miR5477	chr_1:2137385:2137503:-	-26.1	119	0.025	0/0	0/0	6/9	0	6

pti-miR5478	chr_23:97534:97765:+	-76.1	232	0.029	0/0	0/0	5/7	0	4

pti-miR5479	chr_25:444580:444846:+	-79.6	267	0.1	0/0	0/0	18/19	0	4

pti-miR5480	chr_26:278601:278960:+	-105	360	0.146	0/0	0/0	6/8	0	6

pti-miR5481	chr_2:1365749:1366009:+	-70.8	261	0.213	0/0	0/0	5/5	0	4

pti-miR5482	chr_9:128204:128523:+	-101	320	0.561	0/0	0/0	5/6	0	3

pti-miR5483	chr_9:523733:524013:+	-67.6	281	0.126	0/0	0/0	10/12	0	5

^a^Indicated the chromosome number, start and end site and sense (+) or antisense strand (-) that the pre-miRNAs located.

^b^Minimum Free Energy (cal/mol) of pre-miRNAs, predicted by mfold.

^c^length of pre-miRNAs.

^d^computed P-values of miRNA precursors.

^e^number of reads that had the same 5' end as the mature miRNA and the total number reads mapped to the precursors.

^f^asymmetric bulges larger than 2 nt and mismatched miRNA bases in the miRNA region.

### Expression patterns of miRNAs/candidates during nitrogen-limited and silicon-limited conditions, and target prediction

To investigate the probable roles of miRNAs in nitrogen and silicon metabolism in *P. tricornutum*, we sequenced small RNAs from *P. tricornutum *grown in normal, nitrogen-limited and silicon-limited media. Of the 13 miRNAs identified, two appeared in all the three small RNA libraries, one exclusively in PT2 and eight in PT3; and one was shared by PT1 and PT3, and one by PT2 and PT3 (Table 4). The expression of miRNAs in the samples indicated that they might play an important role under nitrogen-limited and/or silicon-limited conditions. To determine the likely regulated genes, we predicted targets of these miRNAs. Using the rules for target prediction suggested by Allen [43], no target was identified. Ignoring locus one and those larger than 21 nt and allowing four mismatches between the miRNA-target duplex in positions 2-21, some potential target sites were suggested (see Additional file 7 for information of potential target genes). Some of these potential targets were involved in lipid metabolism, suggesting that *P. tricornutum *miRNAs might play a role in fatty acid metabolism. This was in accord with the report that *P. tricornutum *accumulated lipids under nitrogen-limited and silicon-limited conditions [32-34]. However, as the genome of *P. tricornutum *is not fully annotated and the functions of many protein-coding genes are unknown, it is difficult to determine whether these miRNA targets have any functional bias.

### siRNA in *P. tricornutum*

It has been reported that in *Arabidopsis*, miRNAs direct the generation of siRNA (termed ta-siRNA), which were phased relatively with each other [43]. To determine whether miRNAs direct the generation of siRNA in *P. tricornutum*, we identified potential siRNAs and determined their location. Potential siRNAs were found in these samples: with 499, 2032 and 2483 unique sequences; and 1206, 6135 and 7836 total sequences in PT1, PT2 and PT3, respectively. The majority of siRNA were produced from a few hot-spots distributed in all the chromosomes; however, they were not phased relatively with each other. To determine whether small RNAs play a role in silencing of repetitive sequences in *P. tricornutum*, as for other organisms, we performed a BLAST search against *P. tricornutum *repeat sequences and found 16 (PT1), 100 (PT2) and 167 (PT3) siRNA derived from these regions. This implied that small RNAs might induce silencing of repetitive sequences in *P. tricornutum*.

### miRNA northern blot

**Figure 3:**
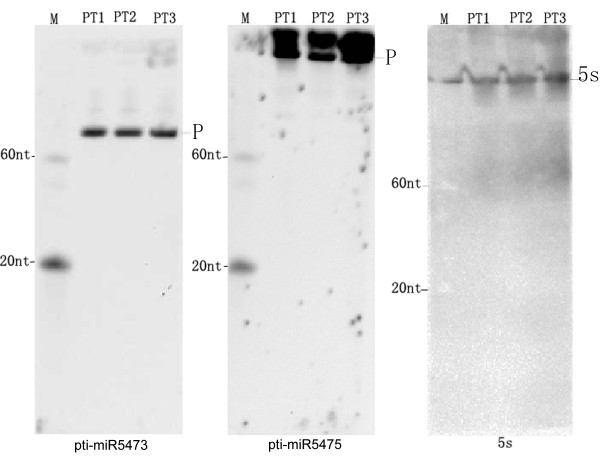
**Northern blot analysis of *P. tricornutum *miRNAs precursors**. Precursors of two miRNAs, pti-miR5473 and pti-miR5475, were detected by northern blotting. 5s RNA was used as load control. M, marker. P, precursor.

MiRNA northern blotting was used to detect initial expression of miRNAs and their precursors in *P. tricornutum*. 5s RNA was blotted as load control. Northern blot hybridization detected precursors of expected size (~100 nt for pti-miR5473 and ~200 nt for pti-miR5475) in all the samples (Figure 3). This provided strong evidence for their expression.

## Discussion

### Did *P. tricornutum *miRNAs evolve independently?

We compared all *P. tricornutum *small RNAs (Table 1) with all known plant, animal and virus miRNAs in miRBase, and found significant identities (Table 5). However, these identities did not pass the criteria we used to identify miRNAs. The most straightforward interpretation for the relative lack of universally conserved miRNAs between *P. tricornutum *and other organisms is that all miRNAs in *P. tricornutum *are rare due to its small genome size, although scenarios that *P. tricornutum *contains novel miRNAs that have no sequence homology with all known ones cannot be ruled out. In a study of miRNAs in the unicellular green alga *Chlamydomonas reinhardtii*, Zhao et al. [40] compared its miRNAs with all known plant and animal miRNAs, and found no homologs. In fact, *C. reinhardtii *lacked homologous miRNAs even with other green algae [40]. Thus we asked whether *P. tricornutum *had some specific miRNAs that have no sequence homology with all known miRNAs, as for *C. reinhardtii*. We predicted novel miRNAs from the small non-annotated RNAs, using the same criteria as used to identify known miRNAs. A total of 13 novel miRNAs were identified from *P. tricornutum *under normal, nitrogen-limited and/or silicon-limited conditions. They lacked homology with all known miRNAs in the miRBase, including *C. reinhardtii *miRNAs. Thus we propose that miRNAs in algae may have evolved independently to animals and plants, consistent with the suggestion of Zhao et al [40].

**Table 5:** The number of known miRNA homologs in *P. tricornutum*.

	Animal		Plant		Virus	
	**Unique**	**Total**	**Unique**	**Total**	**Unique**	**Total**

PT1	54453	2357182	8230	299780	3246	82247

PT2	27865	1470841	4973	121694	1819	45778

PT3	35058	1693263	6732	190221	2270	45259

We also used the *P. tricornutum *chloroplast genome to identify miRNAs. Two loci met all the criteria we used to identify miRNAs. Interestingly, one of these miRNA-like small RNAs was homolog of cin-miR4175, and part of the potential precursor shared 74% identity (21% mismatches and 6.5% gaps) to cin-miR4175 precursor. EST analysis of *P. tricornutum *showed that many of its genes were more similar to animals than photosynthetic organisms [12]. Complete genome sequences showed that diatoms had a mosaic genome with genes from animals, plants and bacteria [13,14]. Thus it is probable that *P. tricornutum *might share some common miRNAs with animals, although the percentage may be relatively low. We propose that this animal miRNA-like small RNA from *P. tricornutum *might be present in diatoms due to gene transformation, or are conserved miRNAs derived from the heterotrophic secondary-host evolutionarily prior to the secondary endosymbiosis, or may be miRNAs lost in the plant/red algal lineage during evolution, similar to the incorporation of animal-like genes in diatoms [13]. If this small RNA found in our study was genuine miRNAs (i.e. *P. tricornutum *contains animal miRNAs, which located in chloroplast genome), then this represents a very interesting discovery.

De Riso, et al. had successfully demonstrated gene silencing in *P. tricornutum *[49]. They analyzed molecular players involved in RNA silencing in *P. tricornutum *and identified both Dicer like proteins (RNA splicing enzyme) and Argonaute like proteins (core components of the effector RNA-induced silencing complexes, RISC). These Argonaute like proteins in *P. tricornutum *clustered in a clade different from either animals or plants [49], suggesting that *P. tricornutum *might own a special RISC pathway different from that of animals and plants, and thus probably result in the lack of preference for U at the 5' of *P. tricornutum *sRNAs.

### Probable roles of miRNAs in metabolism of *P. tricornutum*

miRNAs have been found to play important regulatory roles in various processes in multicellular organisms as well as the unicellular green alga *C. reinhardtii *[18,40]. In the present study, miRNAs were sequenced from *P. tricornutum *under normal, nitrogen-limited and silicon-limited conditions (Table 4). This suggests that miRNAs might play important roles in *P. tricornutum*.

#### miRNAs expressed in all three samples

Two miRNAs appeared in all samples (Table 4). Candidate target genes for these miRNAs included DNA-directed RNA polymerase; glutamate synthase and Δ5 fatty acid desaturase (fatty acid metabolism). This indicates that *P. tricornutum *miRNAs might play important roles in a range of biological processes. It was reported that the composition of fatty acids was significantly influenced by availability of nitrogen [32-34] and silicon [35,36]. Some genes related to glutamate/glutamine metabolism are regulated by silicon availability [38]. Interestingly, we predicted that one gene involved in glutamate synthesis (ferredoxin-dependent glutamate synthase) was targeted by pti-miR5474, which was downregulated in both PT2 and PT3, indicating that miRNA might play a role in silicon-regulated glutamate metabolism.

#### miRNAs that exclusively sequenced from PT3

There were eight miRNAs exclusively sequenced from PT3 (Table 4). Candidate target genes for these miRNAs include phospholipase C isoform delta (lipid metabolic process), nucleotide transporter, ornithine aminotransferase, nucleosome remodeling factor. In *P. tricornutum*, silicification is restricted to one valve of the oval cells and there is no silicon requirement for growth [26]. The strain used in the present study was a fusiform type whose cell wall was not silicified. However, miRNA species were most abundant in PT3 (12/13), and their targets involved in various processes, indicating that various biological processes might be influenced by silicon available through miRNA regulation.

### The enrichment of sRNAs originating from the minus strand of chr13 and both strands of the chloroplast genome

It was interesting that a majority of sRNAs were located in the minus strand of chromosome 13 and both strands of the chloroplast genome (Figure 2). As reported by McFadden and van Dooren [6], green algal/plant and red algal originated from a first endosymbiosis between a eukaryotic and a endosymbiont, whereas diatoms originated from the secondary endosymbiosis between a heterotrophic organism and a red alga. The diatom chloroplast originated from the plasmid of the second endosymbionts, while nucleus of the second endosymbionts lost, living enormous numbers of their genes - typically more than 90% - house in the second host nucleus [6,7,50-52]. We proposed that the enrichment of sRNAs on the minus strand of chr13 as well as both strands of the chloroplast genome indicated that chr13 might have some relative to the second endosymbionts. E.g., chr13 might have originated from nucleus of the second endosymbionts or the majority of the second endosymbionts nuclear genes might have transform into chr13. To test this hypothesis, we extracted the hot spot loci where most small RNA derived from. Those were 39000-46000 nt of the minus strand of chr 13, 63675-70586 nt of the sense strand of chloroplast genome, and 110485-117369 nt of the minus strand of the chloroplast genome. We then aligned them and found that the hot spot locus of chr 13 had no homology with the chloroplast genome. Thus, even if chr 13 have some relative to the second endosymbionts, our data has little support for this hypothesis. We also found that the two hot spot loci of the chloroplast genome in fact share 100% identity. They are two inverted repeats, IRa and IRb, on the chloroplast genome. Thus, small RNAs might play an important role in silencing of inverted repeat region.

### The failure of detection of mature miRNAs by northern blotting was probable due to their low expression

We detected precursors of expected size for pti-miR5473 and pti-miR5475. In other organisms, precursors were more difficult to detect than mature miRNAs in wild type samples [53,54], probably due to their temporary summation in the cells and convert fast into mature miRNAs. We detected miRNA precursors in all the three samples of *P. tricornutum *easily (Figure 3), implied that diatom might obtain different miRNA processor from other organisms, which made the accumulation of miRNA precursors. Expected sizes for the mature miRNAs were not detected. The most straightforward interpretation for this is the low expression of mature miRNAs in the samples we detected, although scenarios that these miRNAs are not real miRNAs but sequencing artifacts or fragments of a longer transcript cannot be ruled out. More sensitive technology is needed to perform further analysis.

## Conclusions

Our results indicated that *P. tricornutum *owned a complex sRNA processing system. It contained novel miRNAs that have no sequence homology with miRNAs of other organisms and that they might play important regulator roles in *P. tricornutum *metabolism.

## Methods

### Strains and culture conditions

Axenic cultures of *Phaeodactylum tricornutum *were available in our laboratory. Cultures were grown in f/2 medium [55] made with steam-sterilized local seawater supplemented with inorganic nutrients and f/2 vitamins (filter sterilized). Cultures were grown at 20°C under cool white fluorescent lights at 24 μmol.m^-2^.s^-1 ^with a 12-h photoperiod for one week. Then cells were harvested by centrifugation for 10 min at 4000 *g*, washed with sterilized seawater, aliquoted into a 500-mL conical flask and then incubated in normal, nitrogen-free and silicon-free f/2 media made with artificial seawater [56] for 48 h. Then cells were harvested by centrifugation for 10 min at 4000 *g*, washed with 4 mL of sterilized seawater, aliquoted into 1.5-mL Eppendorf tubes, and pelleted for 2 min at 10 000 *g*. Cell pellets were frozen instantly in liquid nitrogen and stored at -80°C before RNA extraction.

### Small RNA library construction and sequencing

Total RNA was extracted from *Phaeodactylum tricornutum *cells using the Trizol method according to manufacturer's protocol (Invitrogen, USA). Basically, sRNAs were separated by size fractionation on denaturing polyacrylamide gels. Fragments of 18-28 nt were gel-purified then ligated to a 5'-adaptor and a 3'-adaptor and then RT-PCR-amplified using SuperScript II Reverse Transcription Kit (Invitrogen, USA). RT-PCR product was then sequenced directly using a Solexa 1G Genome Analyzer according to the manufacturer's protocols (see Additional file 1 for flow chart of the procedure for sample preparation and sequencing).

### Initial processing of reads

After removing adaptor sequences and filtering the low-quality tags from the raw reads, the remaining small RNA sequences (clean reads) were mapped to the *Phaeodactylum tricornutum *v2.051706 genome and chloroplast genome [57], using the Short Oligonucleotide Analysis Package (SOAP) [58], all hits were reported and mismatch was not allowed. Non-coding RNAs (rRNA, tRNA, snRNA and snoRNA) degradation fragments were identified by comparing all the clean reads with the sequences of noncoding RNA available in Rfam [59] and the GenBank noncoding RNA database [57], using blastn [60] with a e-value of 0.01 as cutoff. Degraded fragments of mRNA were identified by aligning all the clean reads with exons and introns of mRNAs annotated on the *Phaeodactylum tricornutum *genome and chloroplast genome. sRNAs with perfect overlapped with mRNA sequences were considered as mRNA degraded fragments. homologs of known miRNAs were identified by comparing all the clean reads with the sequences of known miRNAs available from miRBase (miRBase Sequence Database version 15) [42]. If a *Phaeodactylum tricornutum *sRNA exhibited homology with ≤ 2 mismatches (or 90% identity) with other known miRNAs, it was considered a homolog of known miRNAs. Potential siRNA candidates were identified by aligning tags from clean reads to each other; the two perfectly complementary sRNAs with 2 nt hanging at the 3'-end were annotated as siRNA. The remaining sequences were used for further characterization (see Additional file 1 for flow chart of the procedure for processing of reads). All of the raw reads and clean reads generated in this study have been submitted to the GEO at NCBI under accession number GSE29321.

### miRNA identification

After initial processing, homologs of known miRNAs and the remaining non-annotated sRNAs were used to identify miRNAs (see Additional file 1 for flow chart of the procedure for miRNA identification). We first mapped them to genome. sRNAs with more than one read, and ≤ 20 hits to the genome were used for pre-miRNA secondary structure filtering. 300 nt upstream and 300 nt downstream from those loci were extracted and examined for hairpin secondary structures to identify potential miRNAs using criteria developed previously for plant miRNA prediction [43]. Basically, precursors with free energy ≤ -18 kcal/mol checking by Mfold [44,45], ≥ 16 bp and ≤ 4 bulges or asymmetries between miRNA and miRNA*, with miRNA sequence length between 18-25nt and flank sequence length of 20, were considered as potential *Phaeodactylum tricornutum *pre-miRNAs and selected for further analysis. The stabilities of the candidate pre-miRNAs were checked using randfold [61] in dinucleotide shuffling test. Then the 5' homogeneity was checked. The 5' homogeneity was defined as the total number of reads that had the same 5' end as the mature miRNA divide the total number reads mapped to the precursors. For precursors with a low P-value of ≤ 0.05 tested by randfold, a 5' homogeneity >0.5 was applied. For precursors with a P-value > 0.05, a 5' homogeneity ≥0.75 was applied. Then we checked the remaining sequences manually according to criteria made previously [46-48]. Sequences that slightly violated one or none of these primary criteria suggested by each author were obtained.

### miRNA target prediction

The miRanda [62-65] was used to detect potential target sites for the *Phaeodactylum tricornutum *candidate miRNA sequences. The parameters employed were as follows: match score S ≥ 90 and target duplex free energy ΔG ≤ -20 kcal/mol; scaling parameter = 2. The miRNA-target duplexes were then checked manually according to rules suggested by Allen et al. [66] and Schwab et al. [43]. Basically, ≤ 4 mismatches between the small RNA and the target at positions 2-21, counting from the 5' - end of the miRNAs; ≤ 2 adjacent mismatches; no adjacent mismatches in positions 2-12; no mismatches in positions 10-11; and ≤ 2.5 mismatches in positions 1-12 (counting G-U bases as 0.5 mismatches). The minimum free energy (MFE) of the miRNA/target duplex should be >74% of the MFE of the miRNA bound to its perfect complement.

### Northern blotting

The expression of two miRNAs (pti-miR5473 and pti-miR5475) and their precursors were verified by northern blot hybridization using the High Sensitive MiRNA Northern Blot Assay kit (Signosis, USA) according to the manufacturer's protocol. Biotin labeled High Sensitive probe were designed according to the complementary sequences of the mature miRNAs and *Phaeodactylum tricornutum *5s rRNA. 5 *μ*g total RNA was loaded to each well.

## Authors' contributions

AYH carried out the experiments, performed the data analysis and drafted the manuscript. LWH cultured the *P. tricornutum*, prepared the samples and participated in data analysis. GCW conceived of the study, and drafted the manuscript. All authors read and approved the final manuscript.

## Supplementary Material

Additional file 1 **Flow chart of the procedure for sample preparation and sequencing, processing of reads and miRNA identification**. (A) Flow chart of the procedure for sample preparation and sequencing. (1) *P. tricornutum *log phase cells were incubated in normal, nitrogen limited and silicon limited medium for 48 h and harvested, frozen instantly in liquid nitrogen and stored at -80°C before RNA extraction. (2) Total RNA was extracted using the Trizol method. (3) Fragments of 18-28 nt were gel-purified. (4) A 3' adaptor was ligated to the 3' end of sRNAs. (5) A 5' adaptor was ligated to the 5' end of sRNAs. (6) sRNAs were RT-PCR-amplified. (7) Sequencing. (B) Flow chart of the procedure for processing of reads. The numbers in parentheses represented the total reads from PT1, PT2 and PT3, respectively. (1) Initial processing: remove adapter, filter low quality tags and clean up tags smaller than 18nt. (2) Common/specific tags identified between samples. (3) Length distribution analysis of clean reads. (4) Matched clean reads to *P. tricornutum *nuclear genome using SOAP. (5) Matched clean reads to *P. tricornutum *chloroplast genome using SOAP. (6) Compared clean reads with non-coding RNAs from GenBank and Rfam. (7) Exon/intron fragment identified. (8) siRNA identified. (9) Plant miRNA homologs identified. (10) Annotated sRNAs. (11) Identified miRNA by hairpin structure filtering. (12) Target prediction. (C) Flow chart of the procedure for miRNA identification. (a) mfold was used to predict the secondary structure of extracted sequences. Sequences with Δ G < -18 kcal/mol, ≥ 16 bp and ≤ 4 bulges or asymmetries between miRNA and the other arm, miRNA sequence length between 18-25nt, with flank sequence length of 20, were obtained for further analysis. (b) randfold was used to check the stabilities of the candidate pre-miRNAs. (c) 5' homogeneity was checking. For precursors with a low P-value of ≤ 0.05 tested by randfold, a 5' homogeneity >0.5 was applied. For precursors with a P-value > 0.05, a 5' homogeneity ≥0.75 was applied. (d) Criteria made previously for miRNA identification were used to check the remaining sequences manually. Click here for file

Additional file 2 **Nucleotide bias at each position for total small RNA**. The percentages of each type of bases in positions 1 to 24 were indicated by the area. (A) PT1. (B) PT2. (C) PT3. Click here for file

Additional file 3 **Categorization of *P. tricornutum *small RNAs**. The proportion of unique/total sRNA tags matched to all categories of RNAs were showed. (A1) Categorization of unique small RNAs in PT1. (B1) Categorization of unique small RNAs in PT2. (C1) Categorization of unique small RNAs in PT3. (A2) Categorization of total small RNAs in PT1. (B2) Categorization of total RNAs in PT2. (C2) Categorization of total small RNAs in PT3. Click here for file

Additional file 4 **Common and specific sequences between samples**. The common and specific tags of every two samples, including the unique tags and total tags were summarized. (A1) unique sequences of PT1 & PT2. (B1) unique sequences of PT1 & PT3. (C1) unique sequences of PT2 & PT3. (A2) total sequences of PT1 & PT2. (B2) total sequences of PT1 & PT3. (C2) total sequences of PT2 & PT3. Click here for file

Additional file 5 **Patterns of reads mapped to pre-miRNAs**. Click here for file

Additional file 6 **Stem loops for pre-miRNAs**. Click here for file

Additional file 7 **Candidate targets for *P. tricornutum *miRNAs**. Click here for file
