# Supplementary figures and images for: Identification and characterization of microRNAs from Phaeodactylum tricornutum by high-throughput sequencing and bioinformatics analysis
Source: BMC Genomics. 2011 Jun 30;12:337. doi: 10.1186/1471-2164-12-337 (PMC3141676; doi:10.1186/1471-2164-12-337)

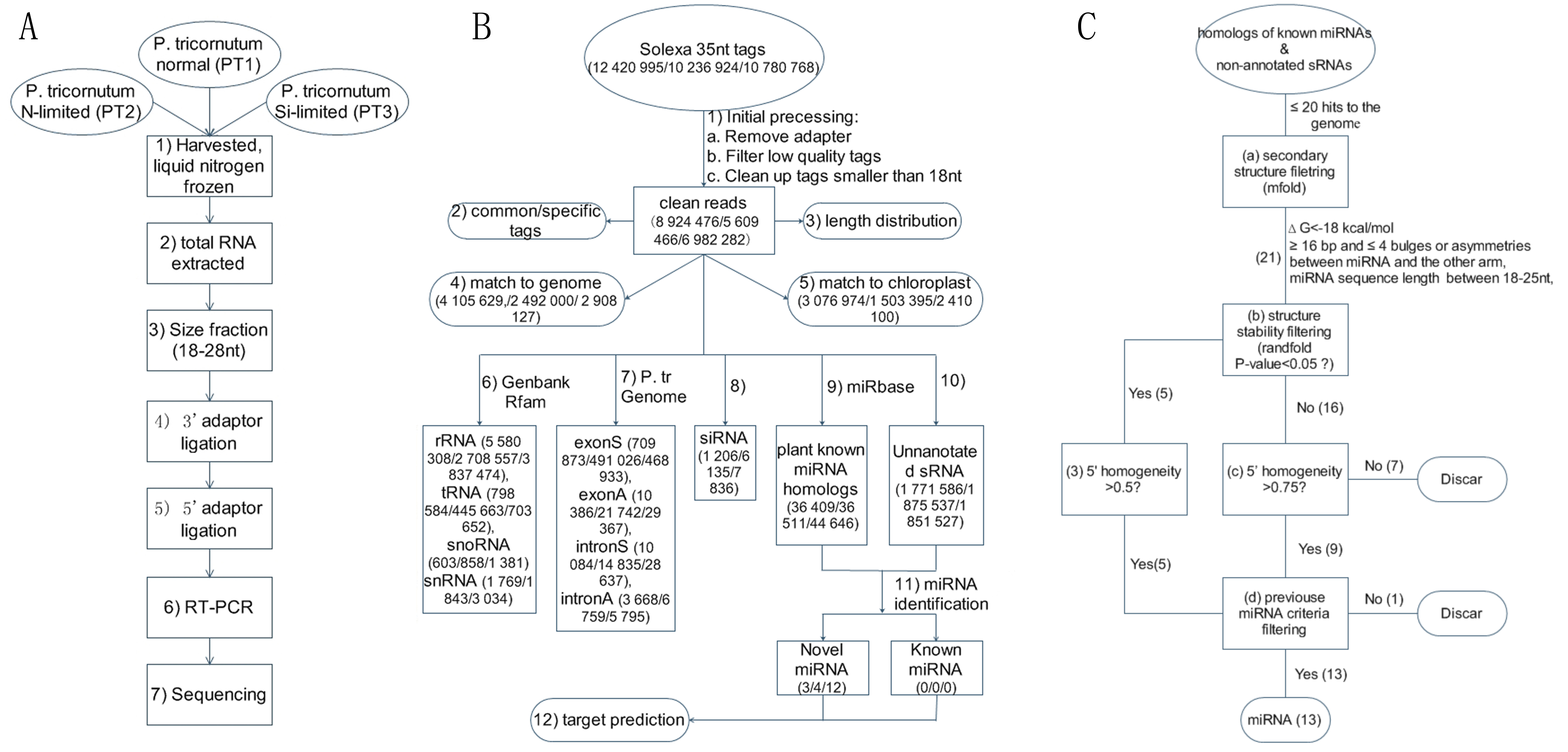

Supplement: Additional file 1 — Flow chart of the procedure for sample preparation and sequencing, processing of reads and miRNA identification. (A) Flow chart of the procedure for sample preparation and sequencing. (1) P. tricornutum log phase cells were incubated in normal, nitrogen limited and silicon limited medium for 48 h and harvested, frozen instantly in liquid nitrogen and stored at -80°C before RNA extraction. (2) Total RNA was extracted using the Trizol method. (3) Fragments of 18-28 nt were gel-purified. (4) A 3' adaptor was ligated to the 3' end of sRNAs. (5) A 5' adaptor was ligated to the 5' end of sRNAs. (6) sRNAs were RT-PCR-amplified. (7) Sequencing. (B) Flow chart of the procedure for processing of reads. The numbers in parentheses represented the total reads from PT1, PT2 and PT3, respectively. (1) Initial processing: remove adapter, filter low quality tags and clean up tags smaller than 18nt. (2) Common/specific tags identified between samples. (3) Length distribution analysis of clean reads. (4) Matched clean reads to P. tricornutum nuclear genome using SOAP. (5) Matched clean reads to P. tricornutum chloroplast genome using SOAP. (6) Compared clean reads with non-coding RNAs from GenBank and Rfam. (7) Exon/intron fragment identified. (8) siRNA identified. (9) Plant miRNA homologs identified. (10) Annotated sRNAs. (11) Identified miRNA by hairpin structure filtering. (12) Target prediction. (C) Flow chart of the procedure for miRNA identification. (a) mfold was used to predict the secondary structure of extracted sequences. Sequences with Δ G < -18 kcal/mol, ≥ 16 bp and ≤ 4 bulges or asymmetries between miRNA and the other arm, miRNA sequence length between 18-25nt, with flank sequence length of 20, were obtained for further analysis. (b) randfold was used to check the stabilities of the candidate pre-miRNAs. (c) 5' homogeneity was checking. For precursors with a low P-value of ≤ 0.05 tested by randfold, a 5' homogeneity >0.5 was applied. For precursors with a P-value > 0.05, [file 1471-2164-12-337-S1.JPEG]

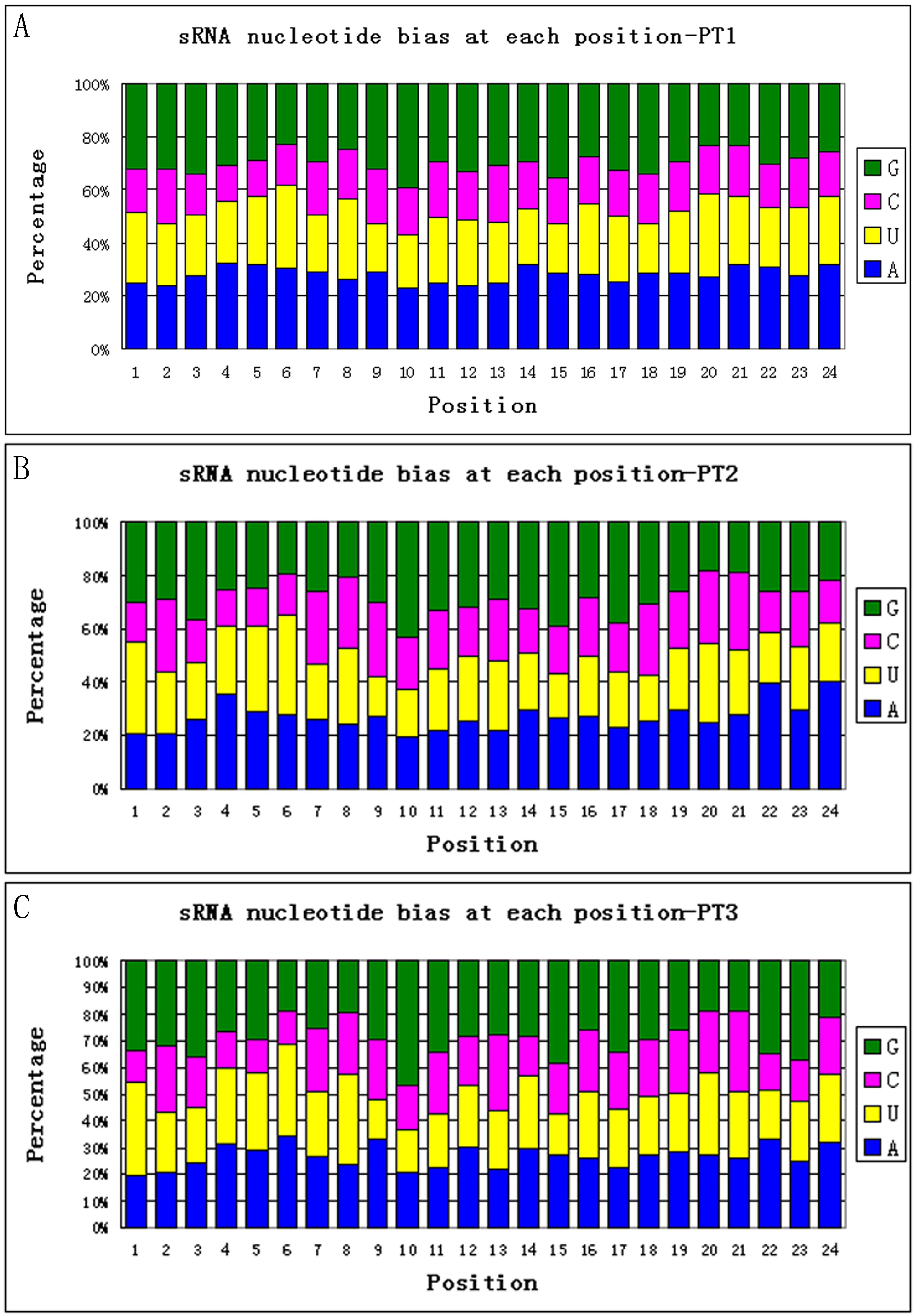

Supplement: Additional file 2 — Nucleotide bias at each position for total small RNA. The percentages of each type of bases in positions 1 to 24 were indicated by the area. (A) PT1. (B) PT2. (C) PT3. [file 1471-2164-12-337-S2.JPEG]

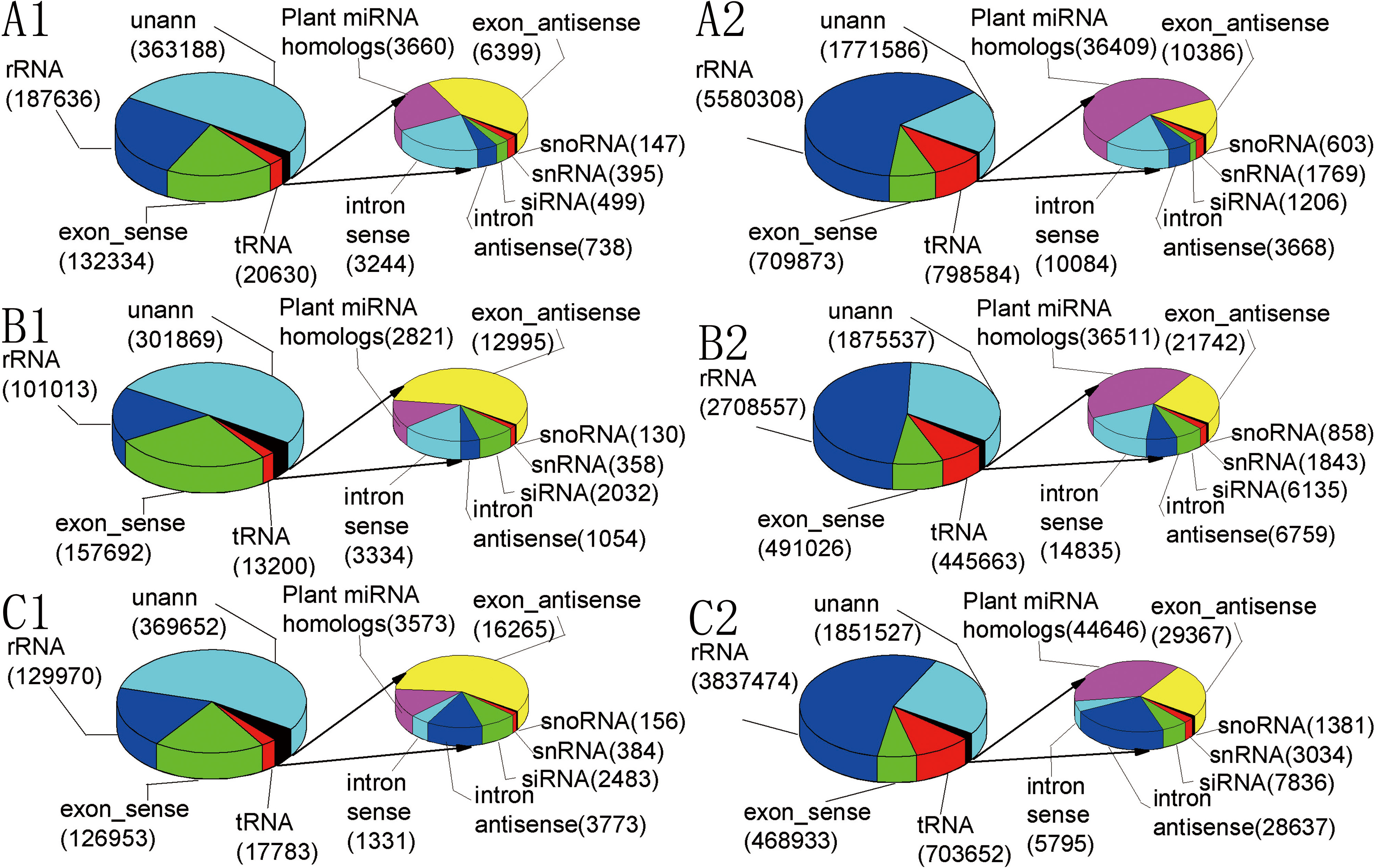

Supplement: Additional file 3 — Categorization of P. tricornutum small RNAs. The proportion of unique/total sRNA tags matched to all categories of RNAs were showed. (A1) Categorization of unique small RNAs in PT1. (B1) Categorization of unique small RNAs in PT2. (C1) Categorization of unique small RNAs in PT3. (A2) Categorization of total small RNAs in PT1. (B2) Categorization of total RNAs in PT2. (C2) Categorization of total small RNAs in PT3. [file 1471-2164-12-337-S3.JPEG]

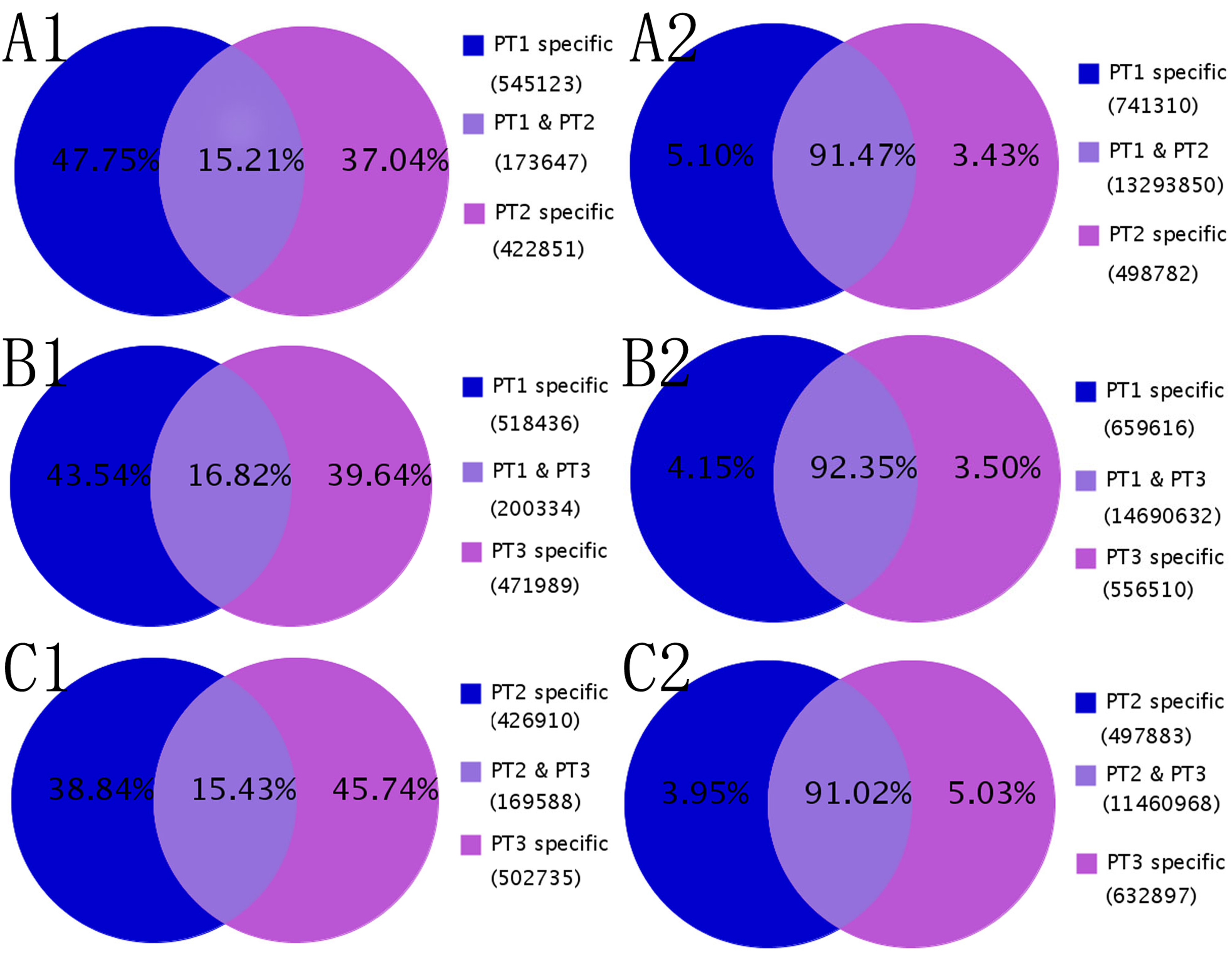

Supplement: Additional file 4 — Common and specific sequences between samples. The common and specific tags of every two samples, including the unique tags and total tags were summarized. (A1) unique sequences of PT1 & PT2. (B1) unique sequences of PT1 & PT3. (C1) unique sequences of PT2 & PT3. (A2) total sequences of PT1 & PT2. (B2) total sequences of PT1 & PT3. (C2) total sequences of PT2 & PT3. [file 1471-2164-12-337-S4.JPEG]

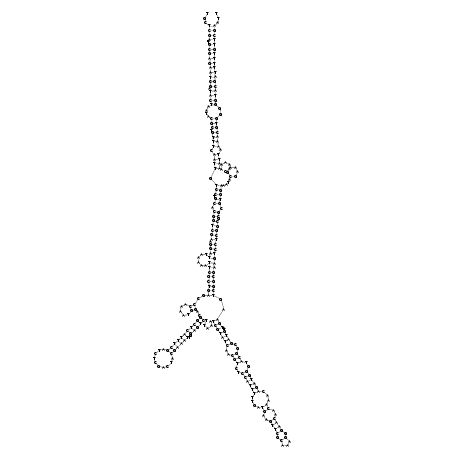


pti-miR5471


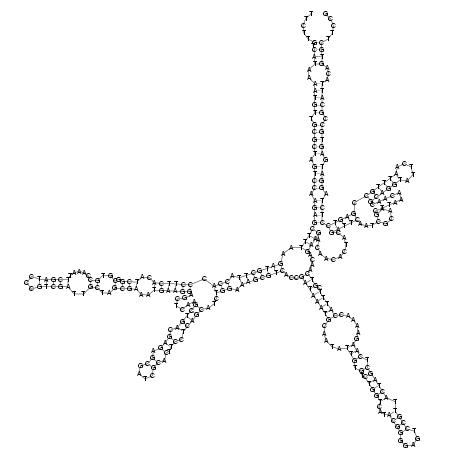


pti-miR5472


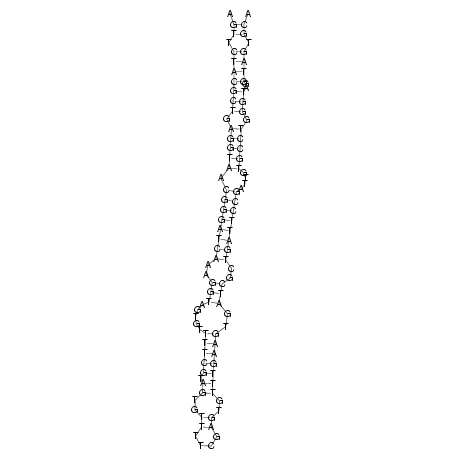


pti-miR5473


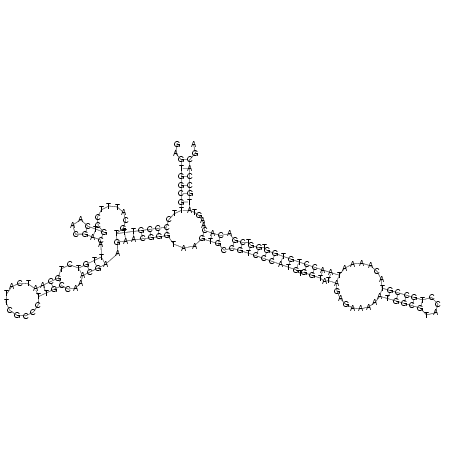


pti-miR5474


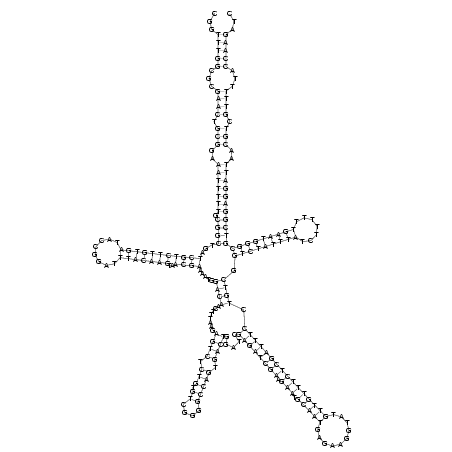


pti-miR5475


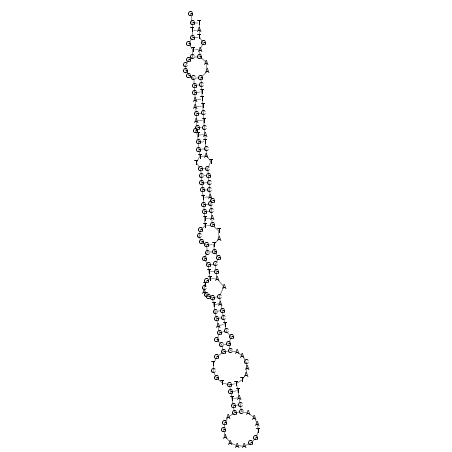


pti-miR5476


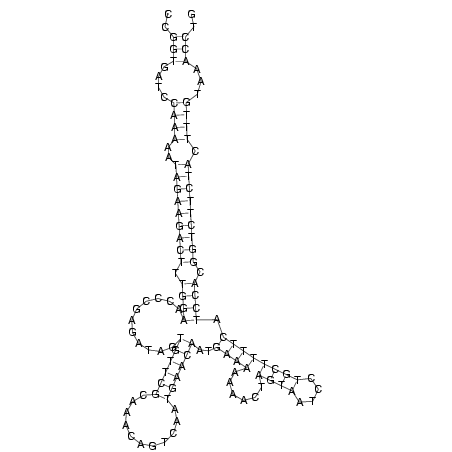


pti-miR5477


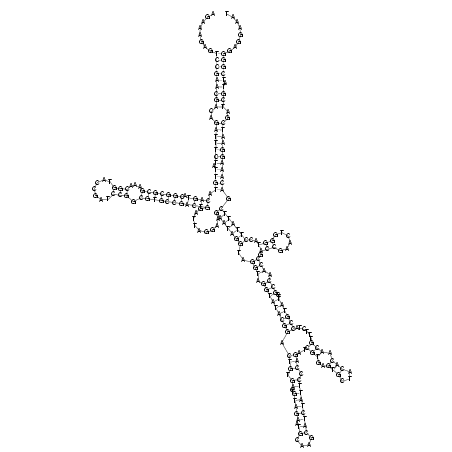


pti-miR5478


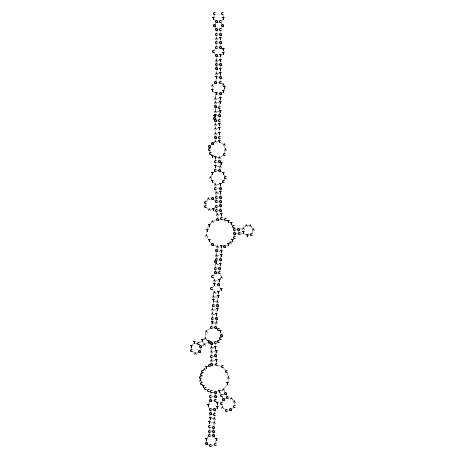


pti-miR5479


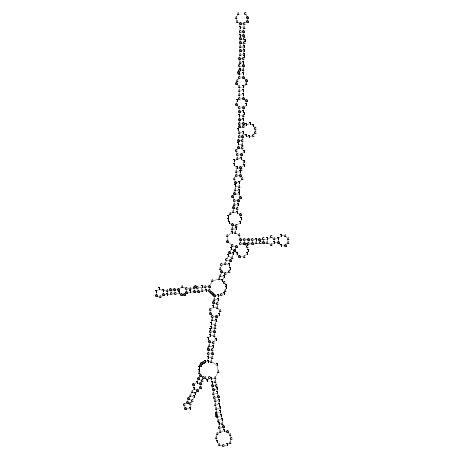


pti-miR5480


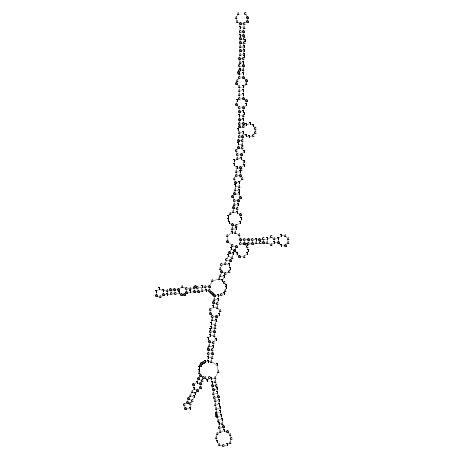


pti-miR5481


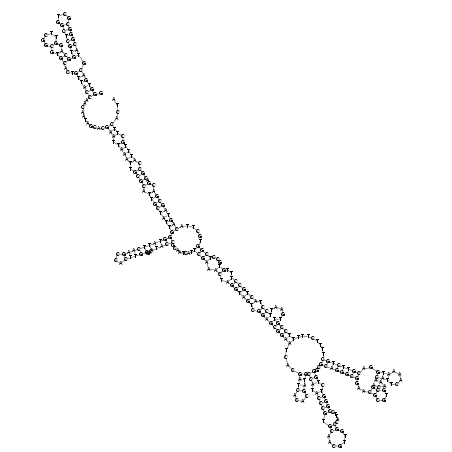


pti-miR5482


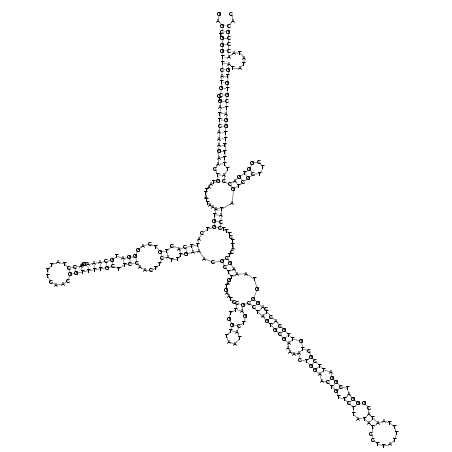


pti-miR5483

Supplement: Additional file 6 — Stem loops for pre-miRNAs. [file 1471-2164-12-337-S6.DOC]
